# Nanoscale electro-structural characterisation of ohmic contacts formed on p-type implanted 4H-SiC

**DOI:** 10.1186/1556-276X-6-158

**Published:** 2011-02-21

**Authors:** Alessia Frazzetto, Filippo Giannazzo, Raffaella Lo Nigro, Salvatore Di Franco, Corrado Bongiorno, Mario Saggio, Edoardo Zanetti, Vito Raineri, Fabrizio Roccaforte

**Affiliations:** 1 Consiglio Nazionale delle Ricerche-Istituto per la Microelettronica e Microsistemi-Strada VIII, n. 5, Zona Industriale, 95121, Catania, Italy; 2 STMicroelectronics, Stradale Primosole 50, 95121, Catania, Italy; 3 Scuola Superiore di Catania, University of Catania, Via Valdisavoia, 9, 95123, Catania, Italy

## Abstract

This work reports a nanoscale electro-structural characterisation of Ti/Al ohmic contacts formed on p-type Al-implanted silicon carbide (4H-SiC). The morphological and the electrical properties of the Al-implanted layer, annealed at 1700°C with or without a protective capping layer, and of the ohmic contacts were studied using atomic force microscopy [AFM], transmission line model measurements and local current measurements performed with conductive AFM.

The characteristics of the contacts were significantly affected by the roughness of the underlying SiC. In particular, the surface roughness of the Al-implanted SiC regions annealed at 1700°C could be strongly reduced using a protective carbon capping layer during annealing. This latter resulted in an improved surface morphology and specific contact resistance of the Ti/Al ohmic contacts formed on these regions. The microstructure of the contacts was monitored by X-ray diffraction analysis and a cross-sectional transmission electron microscopy, and correlated with the electrical results.

## Introduction

Silicon carbide (SiC) is surely the most attractive among the wide band gap semiconductors for the fabrication of high-power and high-temperature electronic devices [1,2].

Ion implantation is the most commonly used technique for selective doping during the fabrication of electronic devices in SiC [3]. In fact, doping of SiC by conventional diffusion techniques cannot be achieved due to the small diffusion coefficients of impurities in the material. Phosphorous implantation is typically used for n-type doping, and an almost complete electrical activation of the dopants can be achieved already at 1500°C [4]. On the other hand, Al implantation is used for p-type doping of SiC [5], and it is typically followed by annealing at higher temperatures (*T *= 1600°C to 1800°C) to promote the electrical activation of the dopant in substitutional lattice sites [6]. However, efficient p-type doping by Al implantation is difficult due both to the high ionisation energies of acceptors and to the high thermal budget required to achieve the electrical activation of the implanted dopants [7]. In particular, the fraction of implanted Al atoms occupying a substitutional position in the SiC lattice has been determined as a function of the post-implantation annealing conditions [8,9]. Furthermore, it has been found that the annealing processes of implanted SiC layers at such high temperatures can induce a significant surface roughness [9]. This roughness in turn can be detrimental for the behaviour of metal/SiC interfaces formed on the implanted regions. All these aspects are still the object of intense discussion in the scientific community working on SiC [5-7,9,10].

In this context, only a scientific approach involving innovative nanoscale techniques can be the way to further improve the basic knowledge on these crucial aspects related to surfaces and interfaces, which can be ultimately useful both for the material growers and for the device makers.

To date, a variety of metals have been used to form ohmic contacts to p-type SiC, the metallisation schemes based on Ti/Al layers being the most promising in terms of specific contact resistance, both on epitaxial and implanted layers [11-14]. However, in these studies, the impact of the surface morphology (influenced by post-implantation annealing conditions) on the properties of ohmic contacts on implanted 4H-SiC was not addressed.

In this work, a nanoscale electro-structural characterisation of 4H-SiC p-type implanted regions was performed, correlating the surface morphology of implanted/annealed SiC with the structural and electrical properties of Ti/Al Ohmic contacts formed on these regions.

## Experimental details

N-type 4H-SiC epitaxial layers, 6 μm thick with a doping concentration of 1.0 × 10^16 ^cm^-3^, grown on heavily doped (8.2 × 10^18 ^cm^-3^) n^+^-type substrate, were used in this work. 'Hot implantation' of the samples was performed at normal incidence at 400°C with Al^+ ^ions using multiple-beam energies (30 to 80 keV) at a dose of 1.3 × 10^15 ^cm^-2 ^to form an almost uniform dopant profile (with an Al concentration of 1 × 10^20 ^cm^-3^). The profile extends over a depth of 175 nm, according to TRIM simulations. Post-implantation annealing for 30 min at 1700°C was carried out for electrical activation of the dopant, *with *and *without *a protective carbon capping layer on the sample surface [15]. The capping layer was removed after the high-temperature annealing. The metal contacts on the p-type Al^+^-implanted regions were formed by a Ti(100 nm)/Al(300 nm) bilayer. The bilayer was deposited by magnetron sputtering and was subjected to rapid annealing at 950°C for 60 s. The specific contact resistance and the sheet resistance of the implanted layers were measured by transmission line model [TLM] structures. The reported results are the average of several measurements performed on ten different patterns fabricated in various regions of the sample surface.

The surface morphology and local electrical properties of both ohmic contacts and implanted layers were investigated by atomic force microscopy [AFM] and conductive AFM [C-AFM]. The microstructural analysis at the ohmic contact metals/4H-SiC was performed using X-ray diffraction [XRD] and transmission electron microscopy [TEM] analyses.

## Result and discussion

**Figure 1:**
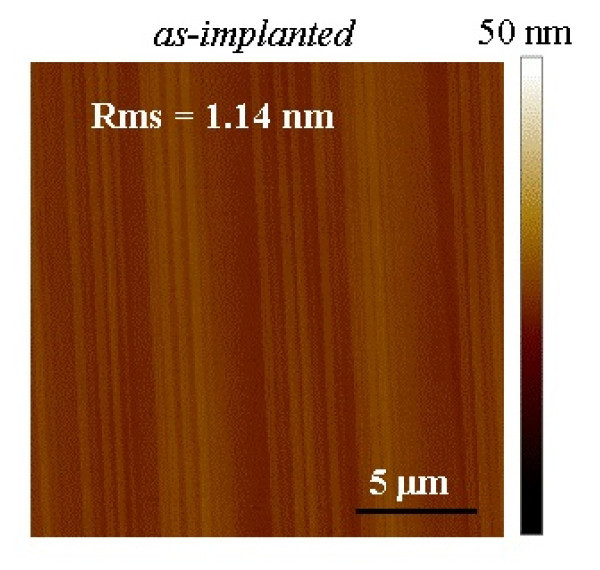
**AFM image of the Al**^**+**^**-implanted 4H-SiC surface "as-implanted" sample**. Scan area of 20 × 20 μm^2^.

First, AFM was used to monitor the morphological properties of the Al^+^-implanted regions. An AFM image taken over an area of 20 × 20 μm^2 ^for the 'as-implanted' sample is reported in Figure 1. As can be seen, before annealing at high temperature, the as-implanted sample exhibits a quite flat surface, with a mean surface roughness value (root mean square, RMS) of 1.14 nm. It is worth noting that this RMS value is comparable to that of the mean surface roughness in 'non-implanted' samples.

**Figure 2:**
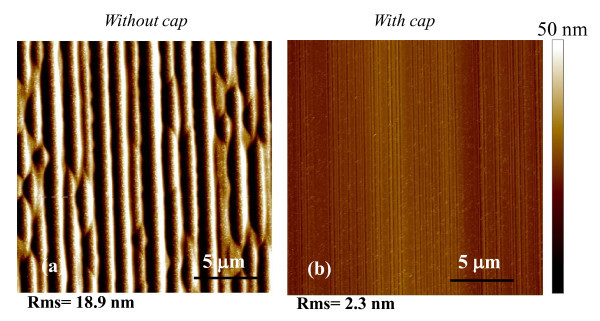
**AFM images of the Al**^**+**^**-implanted and annealed 4H-SiC surface**. (**a) **Sample annealed at 1700°C *without *a protective carbon capping layer. (**b) **Sample annealed at 1700°C *with *a protective carbon capping layer.

Figure 2 shows the AFM scans taken over the same area of 20 × 20 μm^2 ^for the sample annealed at 1700°C *without *or *with *a protective capping layer (Figure 2a,b). While after high-temperature thermal treatment a significant increase of the surface roughness occurs in the sample annealed *without *capping layer, determined by the appearance of the typical step bunching on the surface (Figure 2a), the morphology of the sample annealed *with *a capping layer (Figure 2b) does not exhibit such a surface degradation, and only a slight increase of the roughness with respect to the as-implanted sample is observed. In particular, values of the RMS for the samples annealed at 1700°C *without *and *with *the protective carbon capping layer were 18.9 and 2.3 nm, respectively.

**Figure 3:**
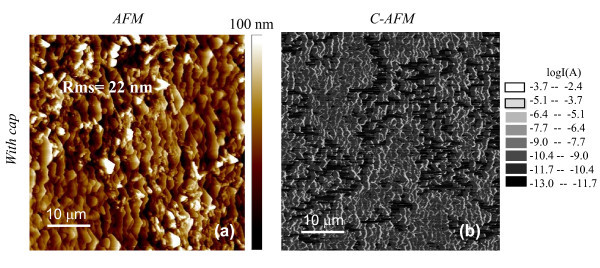
**AFM images and C-AFM current map for samples annealed *with *a protective carbon capping layer**. Surface morphology (**a**) and C-AFM current map (**b**) on a contact Ti/Al fabricated on the Al^+^-implanted 4H-SiC surface and annealed at 1700°C *with *a protective carbon capping layer.

**Figure 4:**
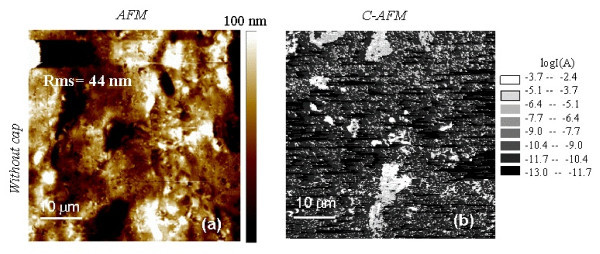
**AFM images and C - AFM current map for samples annealed *without *a protective carbon capping layer**. Surface morphology (**a**) and C-AFM current map (**b**) on a contact Ti/Al fabricated on the Al^+^-implanted 4H-SiC surface and annealed at 1700°C *without *a protective carbon capping layer.

The morphology of the Ti/Al ohmic contacts formed on these implanted regions and their local electrical properties were evaluated by AFM and C-AFM. Figures 3a and 4a show the AFM images for the samples annealed *with *and *without *a protective carbon capping layer, respectively. As can be seen, the Ti/Al annealed contacts are characterised by a high surface roughness, which in turn can be associated with the original morphology of the underlying SiC. In fact, the higher the original SiC surface roughness, the higher is the roughness of the annealed Ti/Al contacts formed on the top of this region. The RMS values of the Ti/Al annealed contacts, deduced from the AFM images, were 22 and 44 nm, respectively, for the sample annealed with and without the capping layer.

The local electrical properties of the contacts were monitored by current measurements performed with C-AFM. This technique provides the maps of the current flowing between the AFM tip in contact with the sample surface and the sample backside and can give information about the local resistance and nanoscale electrical homogeneity of the samples. Two representative current maps on the contacts fabricated on implanted SiC annealed with and without capping layer are reported in the Figures 3b and 4b, respectively (the corresponding morphologies are reported in the Figures 3a and 4a).

As can be seen by the local current maps, a larger inhomogeneity in the local current transport is observed in the Ti/Al ohmic contact formed on SiC annealed without a capping layer.

Macroscopic TLM measurements performed in both samples gave on average similar values of specific contact resistance, *ρ*_*c*_, in the low 10^-4^-Ω cm^2 ^range. In particular, in the sample annealed with a capping layer, *ρ*_c _= 4.90 × 10^-4 ^Ω cm^2^, whilst in the uncapped one, *ρ*_c _= 3.67 × 10^-4 ^Ω cm^2^. However, it must be pointed out that a significant dispersion of the values of *ρ*_c _extracted by TLM measurements was observed, particularly in the uncapped sample. This dispersion can be ascribed either to the different degrees of local electrical inhomogeneity of the metal/SiC contact oberved by C-AFM or to an inhomogeneous dopant activation over the sample surface (whose sheet resistance was in the order of 10^4 ^Ω/sqr). A similar interpretation on the spread in the values of *ρ*_c _in the case of Ti/Al contacts on p-type ion-implanted SiC was suggested also by other authors [13,16].

The structural properties of the samples were evaluated by XRD analysis combined with a cross-sectional TEM.

The main feature deduced by XRD analysis (not shown here) performed on Ti/Al annealed contacts is the formation of a ternary compound, Ti_3_SiC_2_, independent of the use of a capping layer during activation annealing. The formation of other phases like Al_4_C_3_, already reported in [14], has not been detected in our samples. Furthermore, XRD analysis indicated the presence of a significant amount of unreacted Al, particularly in the sample annealed with a capping layer. In fact, large agglomerates have been observed on the contact surface by optical inspection and AFM analysis. A compositional analysis performed by "energy-filtered TEM [EFTEM] revealed the presence of pure Al. Similar agglomerates were already observed by Parisini et al. [17] in Al-rich Al/Ti-SiC alloyed contacts, and their origin was attributed to the freezing of an excess of liquid Al during the cooling down of the samples after annealing.

**Figure 5:**
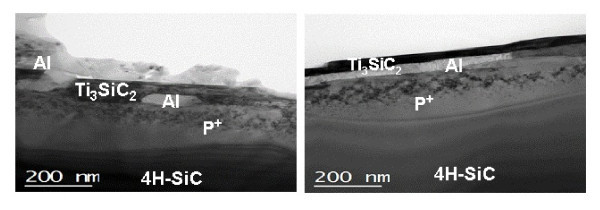
**Bright field cross-section TEM images for Ti/Al ohmic contacts**. Images for Ti/Al contacts annealed without capping layer (**a**) and with capping layer (**b**).

Cross-section TEM analysis allowed us to monitor the different microstructures of the contacts especially in the proximity of the interface. Figure 5a,b shows the cross-sectional TEM micrographs for samples annealed without and with a capping layer, respectively.

EFTEM analysis allowed identifying the elemental composition of the grains inside the reacted metal layer. In particular, the ternary phase Ti_3_SiC_2_, identified by XRD analysis, was confirmed by EFTEM chemical maps. This phase was formed due to the thermal reaction of Ti with SiC at high temperatures [18]. However, whilst in the sample without a capping layer (Figure 5a) large Ti_3_SiC_2 _grains are located close to the interface with SiC and interrupted by small Al-rich regions, in the sample with a capping layer (Figure 5b), larger Al-rich regions are found, in some parts forming an almost continous interfacial layer. These results suggest that the reaction mechanism (and hence the final interfacial microstructure) is affected by the original SiC surface roughness. The higher structural homogeneity of the metal/SiC interface in the sample annealed with the capping layer (characterised by more uniform Ti_3_SiC_2_- and Al-rich regions) is consistent with the macroscopic and nanoscale electrical behaviour observed by TLM and C-AFM measurements, respectively.

## Summary

In this work, an electro-structural characterisation of Ti/Al ohmic contacts formed on p-type Al-implanted 4H-SiC was performed using nanoscale analysis techniques.

The surface morphology of the Al-implanted regions after annealing was analyzed as a function of the different annealing conditions (with and without a capping layer). The significant step bunching occurring in Al-implanted SiC surfaces after high-temperature thermal treatments could be prevented by using a protective carbon capping layer during annealing. Consequently, Ti/Al ohmic contacts fabricated on the implanted regions annealed with a capping layer exhibited a lower surface roughness and better electrical uniformity with respect to the contacts formed on the regions annealed without a capping layer.

The structural analysis of the contacts showed the occurrence of an interfacial reaction between Ti and SiC, with the formation of a ternary compound Ti_3_SiC_2_, and an interfacial microstructure strongly dependent on the roughness of the underlying SiC.

## Competing interests

The authors declare that they have no competing interests.

## Authors' contributions

AF carried out the electrical measurements, performed the electrical analysis and drafted the manuscript. FG carried out the AFM images and C-AFM current maps. RLN carried out the XRD spectra. SDF carried out the lithographic processes. CB carried out the TEM analysis. MS and EZ provided the wafers and carried implants and annealing processes. VR participated in the design of the study and its coordination. FR planned the experiment, participated in its coordination, worked in data interpretation and drafted the manuscript. All authors read and approved the final manuscript.
